# A multiparametric fluorescence assay for screening aptamer–protein interactions based on microbeads

**DOI:** 10.1038/s41598-022-06817-0

**Published:** 2022-02-22

**Authors:** Carsten Schmidt, Anne Kammel, Julian A. Tanner, Andrew B. Kinghorn, Muhammad Moman Khan, Werner Lehmann, Marcus Menger, Uwe Schedler, Peter Schierack, Stefan Rödiger

**Affiliations:** 1 Environment and Natural Sciences, Institute of Biotechnology, Brandenburg Technical University Cottbus-Senftenberg, Universitätsplatz 1, 01968 Senftenberg, Germany; 2 School of Biomedical Sciences, LKS Faculty of Medicine, The University of Hong Kong, 21 Sassoon Road, Pokfulam, Hong Kong; 3 Attomol GmbH, Schulweg 6, 03205 Bronkow, Lipten Germany; 4 Faculty of Health Brandenburg, Brandenburg University of Technology Cottbus-Senftenberg, Senftenberg, Germany; 5 Fraunhofer Institute for Cell Therapy and Immunology, Branch Bioanalytics and Bioprocesses (IZI-BB), Am Mühlenberg 13, 14476 Potsdam, Germany; 6 PolyAn GmbH, Rudolf-Baschant-Straße 2, 13086 Berlin, Germany

**Keywords:** Biochemistry, Biological techniques, Biotechnology, Molecular biology, Biomarkers

## Abstract

For improving aptamer-ligand binding we have developed a screening system that defines optimal binding buffer composition. Using multiplex assays, one buffer system is needed which guarantees the specific binding of all aptamers. We investigated nine peer-reviewed DNA aptamers. Non-specific binding of aptamers is an obstacle. To address this, we investigated 16 proteins as specificity controls bound covalently to encoded microbeads in a multiplex assay. Increasing the NaCl concentration decreased the binding for all aptamers. Changing pH values by one unit higher or lower did not influence the aptamer binding significantly. However, pH < 5 led to non-specific binding for all aptamers. The *Pf*LDH-aptamer selected in the absence of divalent cations exhibited doubling of its binding signal by the addition of Ca^2+^ and Mg^2+^. We confirmed Ca^2+^ and Mg^2+^ dependency of the aptamers for streptavidin and thrombin by observing a 90% and 50% binding decrease, respectively. We also achieved a doubling of binding for the streptavidin aptamer when replacing Ca^2+^ and Mg^2+^ by Mn^2+^. A buffer suitable for all aptamers can have considerable variations in pH or ionic strength, but divalent cations (Ca^2+^, Mg^2+^, Mn^2+^) are essential.

## Introduction

Aptamers were first described by Tuerk and Gold^1^ and are ssDNA or RNA oligonucleotides that have the potential to bind with high affinity and specificity to a target molecule. Hence, aptamers are considered as alternatives to antibodies and are very useful for biosensor applications^2,3^ or as therapeutic agents^4^.

Aptamers are isolated by an in vitro process called “systematic evolution of ligands by exponential enrichment” (SELEX)^5^. During SELEX, an aptamer library is screened for sequences that have an affinity for a given target molecule. Aptamers are an interesting class of affinity reagents that may require extensive modifications to fulfil criteria like affinity, specificity, and therapeutic half-life for a specific clinical need^4,6^. Therefore, high-throughput technologies are needed to test the effects of modified aptamers.

In the literature and after discussions with other experts we recognised several challenges for aptamer development^7^. Their three-dimensional structure, which is essential for target interaction, can be affected by environmental factors such as pH, salt concentrations, and temperature^8^. Therefore, each aptamer must be optimised individually. The physicochemical properties of the aptamers and their molecular targets, such as the thermodynamic interactions between all molecules involved (aptamer ↔ target, aptamer ↔ non-target), the synthesis chemistry (e.g. aptamer modifications with sugars and dyes), and surface physics play a role in defining the aptamer utility.

We have developed the fully automated fluorescence imaging VideoScan platform^9^ to perform multiparametric assays. This technology is based on fluorescence-encoded microbeads, coupled with different capture probes (usually oligonucleotides or antibodies) against certain target molecules. The microbeads are mixed with an analyte solution. If the target molecule is present it will bind to its capture probe and hence to a particular microbead population. To visualise this binding, fluorescence labelled detection probes (e.g. antibodies, oligonucleotides or aptamers) are used, so that a fluorescence halo will appear on the surface of the microbeads. However, this multiplex approach is only functional if all the different antibodies or aptamers are compatible within the same buffer system. Antibodies are usually used in common TBST or PBST buffers at neutral pH. For aptamers the situation is different. Aptamers are screened in the presence of a variety of buffers having different pH, different ionic strengths, while some buffers contain Na^+^, K^+^, Mg^2+^, Ca^2+^ or detergents. Choosing aptamers by looking for published aptamers in the literature will lead to an aptamer list, whose binding buffers are all different. This makes multiplexing of aptamers (using different aptamers in the same buffer) very challenging. The worst case scenario would be the screening of the aptamers by SELEX using the assay buffer as the selection buffer, because establishing and conducting SELEX is time-consuming and difficult. The absence of a common binding buffer for each aptamer is unfavorable and inconvenient. But how important is the exact composition of a binding buffer for aptamer-target interaction? Does an aptamer lose its binding capacity when used in a buffer that is different from the SELEX selection buffer? Do aptamers tolerate changes in the binding buffer composition and can aptamer binding be improved by using a different binding buffer?

We selected nine published aptamers, one each for streptavidin (SA-apta)^10^, interferon γ (IFNγ-apta)^11^, lactate dehydrogenase from *Plasmodium falciparum* (*Pf*LDH-apta)^12^, protein A (PA-apta)^13^, tumor necrosis factor α (TNFα-apta)^14^, enterotoxin B from *S. aureus* (Entero-apta)^15^, mouse IgG (mIgG-apta)^16^ and two for thrombin (T1-apta, T2-apta)^17,18^. The sequences and selection buffers of these aptamers are summarised in Table 1. All have important bioanalytical or pharmaceutical applications. Interferon γ (IFNγ) is a glycoprotein produced by lymphocytes. IFNγ has antitumoral, antiviral and immunomodulatory functions. Therefore, IFNγ assays are widely used in research and clinical diagnosis. Hemophagocytic lymphohistiocytosis (HLH) is a rare, extremely severe hyperinflammatory disease of the immune system. IFNγ is considered a critical factor in the development of the disease. Immuno-chemotherapy, primarily etoposide-based regimens, is currently the only pharmacological approach^19,20^. In recent years, procedures have been discussed that neutralize IFNγ. In principle, aptamers are also suitable in addition to antibodies.

**Table 1:** Selection buffer composition of the used aptamers. *nL* not labelled. Aptamer sequences can be found in Supplementary Table S1.

Aptamer	Target	Original labeling^a^	Selection buffer
T1-apta^17^	Thrombin	^32^P	20 mM Tris/HCl (pH 7.4), 100 mM NaCl, 5 mM KCl, 1 mM MgCl_2_, 1 mM CaCl_2_
T2-apta^18^	Thrombin	^32^P	50 mM Tris/HCl (pH 7.5), 100 mM NaCl, 1 mM MgCl_2_
IFNγ-apta^11^	IFNγ	nL	20 mM Tris (pH 7.6), 100 mM NaCl, 5 mM KCl, 2 mM MgCl_2_, 1 mM CaCl_2,_ 0.02% Tween 20
SA-apta^10^	Streptavidin	5′-Fluorescein	20 mM Tris (pH 7.6), 100 mM NaCl, 5 mM KCl, 2 mM MgCl_2_, 1 mM CaCl_2,_ 0.02% Tween 20
*Pf*LDH-apta^12^	*Pf*LDH	nL	8.1 mM Na_2_HPO_4_, 1.47 mM KH_2_PO_4_, (pH 7.4), 137 mM NaCl, 2.7 mM KCl
PA-apta^13^	Protein A	5′-Fluorescein	20 mM Tris/HCl (pH 7.6), 100 mM NaCl, 5 mM KCl, 10 mM MgCl_2_, 1 mM CaCl_2_, 0.005% Tween 20
TNFα-apta^14^	TNFα	^32^P	100 mM Phosphate (pH 7.0), 150 mM NaCl, 0.005% Tween 20
Entero-apta^15^	*S. aureus* enterotoxin B	^32^P	10 mM Phosphate (pH 7.4), 140 mM NaCl, 2.7 mM KCl, 0.05% Tween 20
mIgG-apta^16^	Mouse IgG	^32^P	8.1 mM Na_2_HPO_4_, 1.47 mM KH_2_PO_4_, (pH 7.4), 137 mM NaCl, 2.7 mM KCl, 5 mM MgCl_2_

^a^We used all sequences labelled with Cy5 at the 5′-end.

We analysed aptamer interaction with their targets by systematically modifying the buffers used for the SELEX or taking completely different buffers. The aptamer targets were coupled to dye/size-encoded microbeads enabling simultaneous analysis of different binding targets with an easy to perform spin down assay. There are various applications of aptamers. In aptamer-based sensor applications, aptamers are often immobilised at solid phases and used to detect molecules. Here it is necessary to consider that the binding behaviour of immobilised aptamers can change. Another typical application is to keep aptamers in solution as sensor molecules, so that they can interact with other molecules. This isthe case in the screening for aptamers with SELEX, or when aptamers are used as substitutes for antibodies in ELISA. This is the case in the screening for aptamers with SELEX, or when aptamers are used as substitutes for antibodies in ELISA where the enzyme-linked apta-sorbent assay (ELASA) is used, the targets are immobilised, and the aptamers are in solution^21^. We consider this case due to its high bioanalytical relevance. In contrast to other studies, we have included microbead populations with proteins in all experiments to which the aptamers are not to bind. Microbead formulations without proteins are also included as negative control. Thus, we were able to test the aptamers simultaneously against multiple negative controls to identify effects on the aptamer specificity. We stress that studies by other authors did not take this into account. With immobilised aptamers, these tests for specificity could not have been carried out as easily as multiplexing would not have been possible.

We were especially interested in the robustness of aptamer binding and hence focused on the question of how difficult it is to find common binding conditions that are optimal for all investigated aptamers. The testing of different binding buffers is costly and becomes even more complex in multiplex assays, in which different aptamers are used in combination. By performing analyses in a 96-well format and multiplexing microbeads, we were able to sample large quantities efficiently and in a cost effective manner.

## Results and discussion

### Aptamer-target binding using a multiplex assay

**Figure 1:**
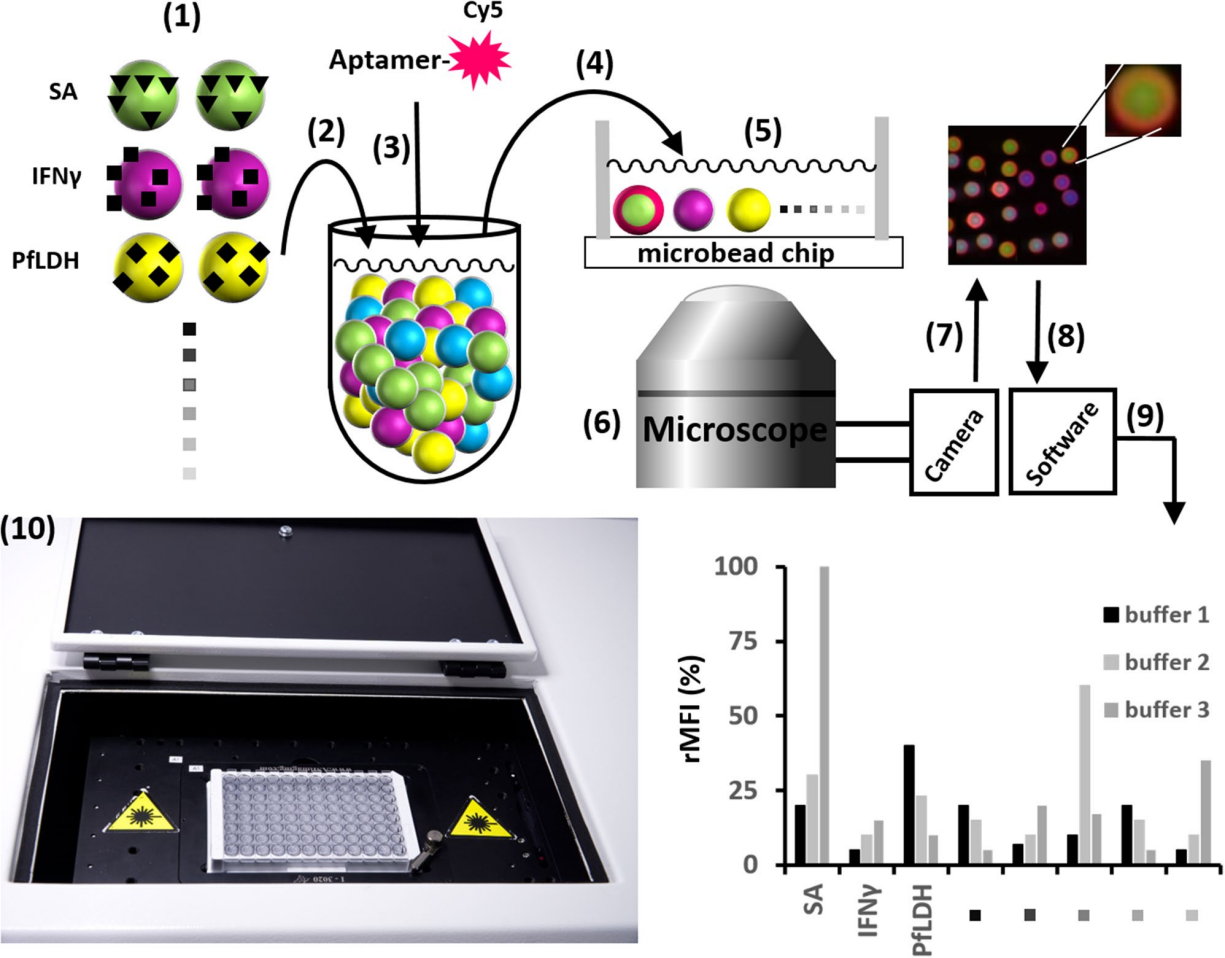
Principle of the multiplexed aptamer binding assay using VideoScan technology. (1) Dye/size-encoded microbead populations presenting different proteins on their surfaces are mixed (2) incubated with a fluorescence-labelled aptamer (3) dissolved in a binding buffer of choice. After removing unbound aptamers by washing, the microbead suspension was transferred into a cavity of a 96 well plate (4). The microbeads were allowed to settle down forming a microbead chip on the transparent bottom (5). A fluorescence microscope (6) is used to take pictures of the microbead chip (7). Imaging software analyses the pictures, recognises and counts microbeads, measures their surface fluorescence intensity and groups them into populations (8). Finally, for each population the referenced mean fluorescence intensity (rMFI) per microbead population is calculated (9). (10) Shows a 96-well microtiter plate placed into the VideoScan system.

Our experimental approach presented in Fig. 1 required dye/size-encoded microbead populations presenting potential aptamer target proteins and some non-target proteins (as specificity controls) on their surfaces. We coupled different proteins via EDC-chemistry to carboxylated microbead populations and checked for successful coupling with appropriate detection probes (Supplementary Table S2). Results are shown in supplementary information (Supplementary Fig. S1).

Due to non-directional binding, the target proteins have random orientation on the surface. The orientation may have an influence on the binding (e.g., inaccessible binding site, deformation) or activity (e.g., catalysed activity)^13,22^. But we argue that the random orientation can also stochastically make accessible a portion that is usually sufficient for the generation of a measurement signal. This is also in line with our previous work^23–25^, where molecules were successfully bound.

**Figure 2:**
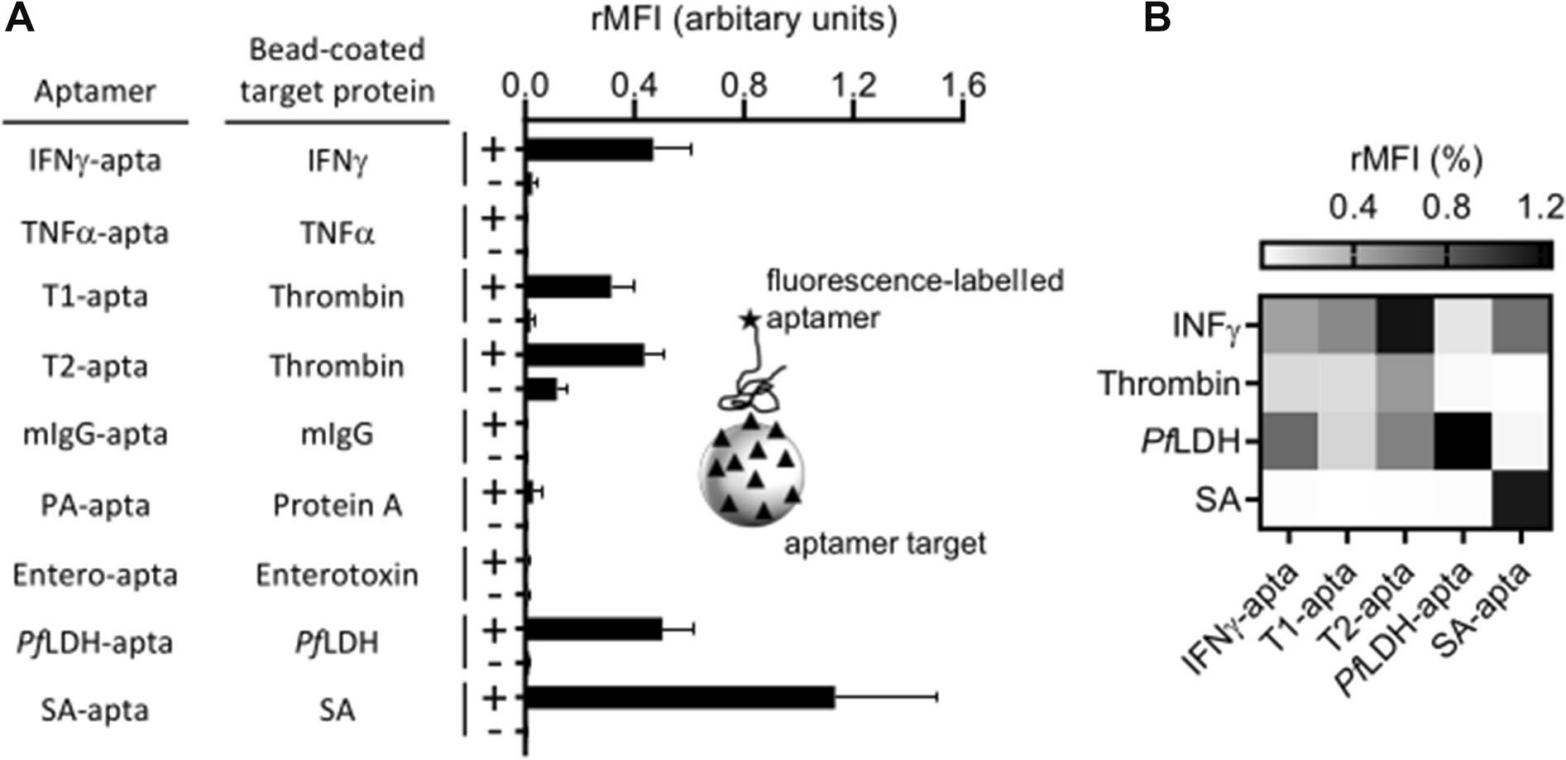
(**A**) Binding functionality of nine aptamers to their targets immobilised on the surface of fluorescence microbeads. (+) indicates microbeads coupled with indicated target and (−) indicates microbeads coupled with ethanolamine as a negative control (**B**) Specificity of aptamer binding against non-target protein within their corresponding selection buffer (Table 1) as binding buffer. Shown are mean ± SD (n = 3–8).

We then examined the binding of the nine aptamers to microbeads presenting their target molecules (Fig. 2A).

We were unable to detect any signal mIgG, although the presence of the corresponding target coupled to the surface of the microbeads was clearly shown by the use of antibodies (Supplementary Fig. S1). Aptamers are considered as a substitute for antibodies, and researchers often assume that aptamers can be modified in a similar way without losing their function. The fluorescence-based measurement is a very sensitive method with applications in many areas. The nine aptamer sequences were taken from original publications of other researchers. We uniformly labelled the aptamers with the water-soluble fluorescent dye sulfo-Cy5, which is a commonly used label in bioanalytics (Table 1). It should be stressed that any modification can change the properties of aptamers, especially the three-dimensional structure^13,26^. The solubility of an aptamer can be increased or decreased depending upon the hydrophilicity of the label. The presence of a label could also block a binding site or prevent the functional three-dimensional folding of an active aptamer. Changing the originally published detection label to Cy-5 may influence the binding of the aptamer to its target and could be one explanation why we did not detect a binding for all selected aptamers. For example, Stoltenburg et al. showed considerably different affinities of the protein A aptamer (PA-apta) depending on the position of a biotin label^13^. We procceded with the five aptamers that gave detectable signals. In this paper the aptamer concentration was generally 500 nM, since at this concentration we saw a saturated binding signal for all aptamers (Supplementary Fig. S2). We observed unspecific binding to non-target proteins (Fig. 2B): The proteins interferon γ, thrombin and *Pf*LDH were bound by different aptamers, whereas streptavidin was only bound by its specific aptamer. Proteins with high isoelectric point (pI) values, like interferon γ and thrombin, have a positive net charge in neutral binding buffer (Supplementary Table S3) and therefore they are liable to make unspecific weak electrostatic binding to DNA in general.

Furthermore, we investigated the effect of boiling and chilling of the aptamers before usage (Supplementary Fig. S3A). We expected a binding increase by heating the aptamers to 95 °C, because this boiling step is part of many SELEX procedures. Supplementary Figure S3A shows that heating and cooling the aptamers did not markedly influence the binding of the target proteins without the aptamer T2-apta.

### Aptamer binding decreased with increasing ionic strength

**Figure 3:**
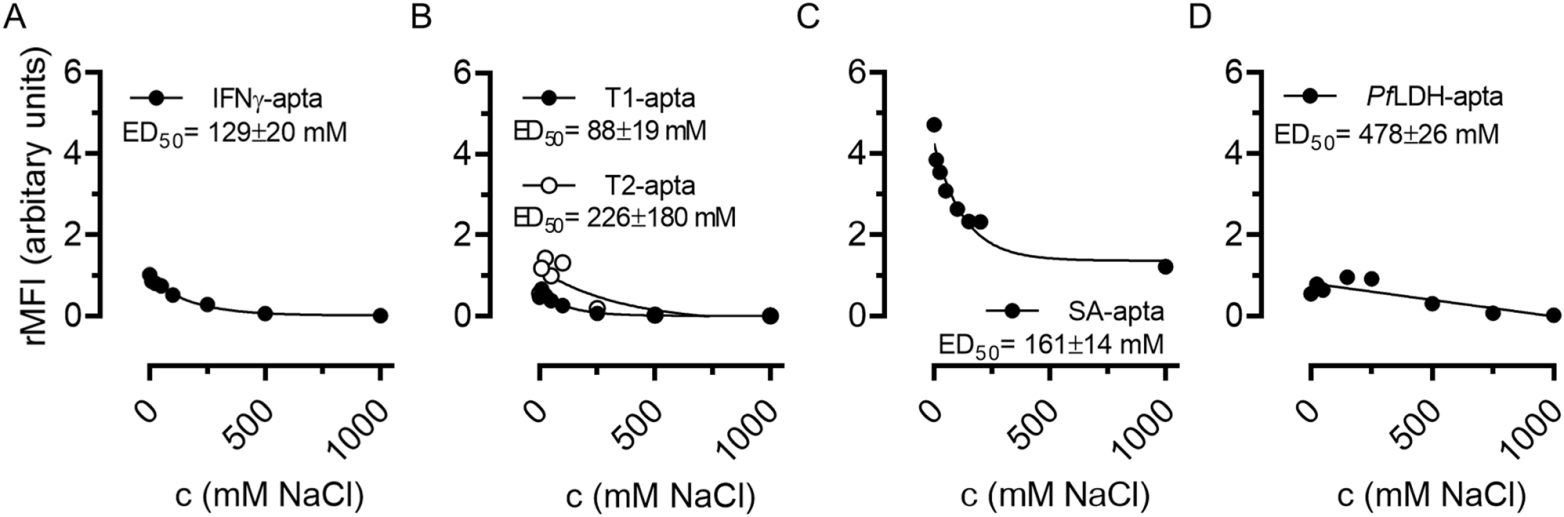
Influence of ionic strength on aptamer binding. Microbeads coupled with (**A**) IFNγ, (**B**) thrombin, (**C**) streptavidin and (**D**) *Pf*LDH were incubated with their fluorescence-labelled aptamers in the presence of varying NaCl concentrations (0–1000 mM). The binding of the aptamer to its target was measured by quantifying the surface fluorescence of the microbeads using VideoScan technology (mean values, n = 2). The half-maximal value (ED_50_ in mM) was calculated after fitting non-linear models (IFNγ-apta: EXD.2; T2-apta: LL.2; T1-apta, SA-apta, *Pf*LDH-apta: LL.3).

In order to investigate how sensitive the aptamer binding was upon variation of ionic strengths, we increased the NaCl concentration of each binding buffer systematically. The results show that the aptamer binding is primarily mediated via electrostatic forces since an increasing NaCl concentration leads to decreasing aptamer binding forces since an increasing NaCl concentration leads to decreasing aptamer binding. (Fig. 3).

We increased the NaCl concentration of each binding buffer systematically. The results show that the aptamer binding is primarily mediated via electrostatic forces since an increasing NaCl concentration leads to decreasing aptamer binding. The greatest binding signals were obtained in the absence of NaCl. However, for *Pf*LDH-apta we saw an optimal NaCl concentration of about 150 mM (Fig. 3D).

### At pH values below 5, aptamers bind completely non-specifically

**Figure 4:**
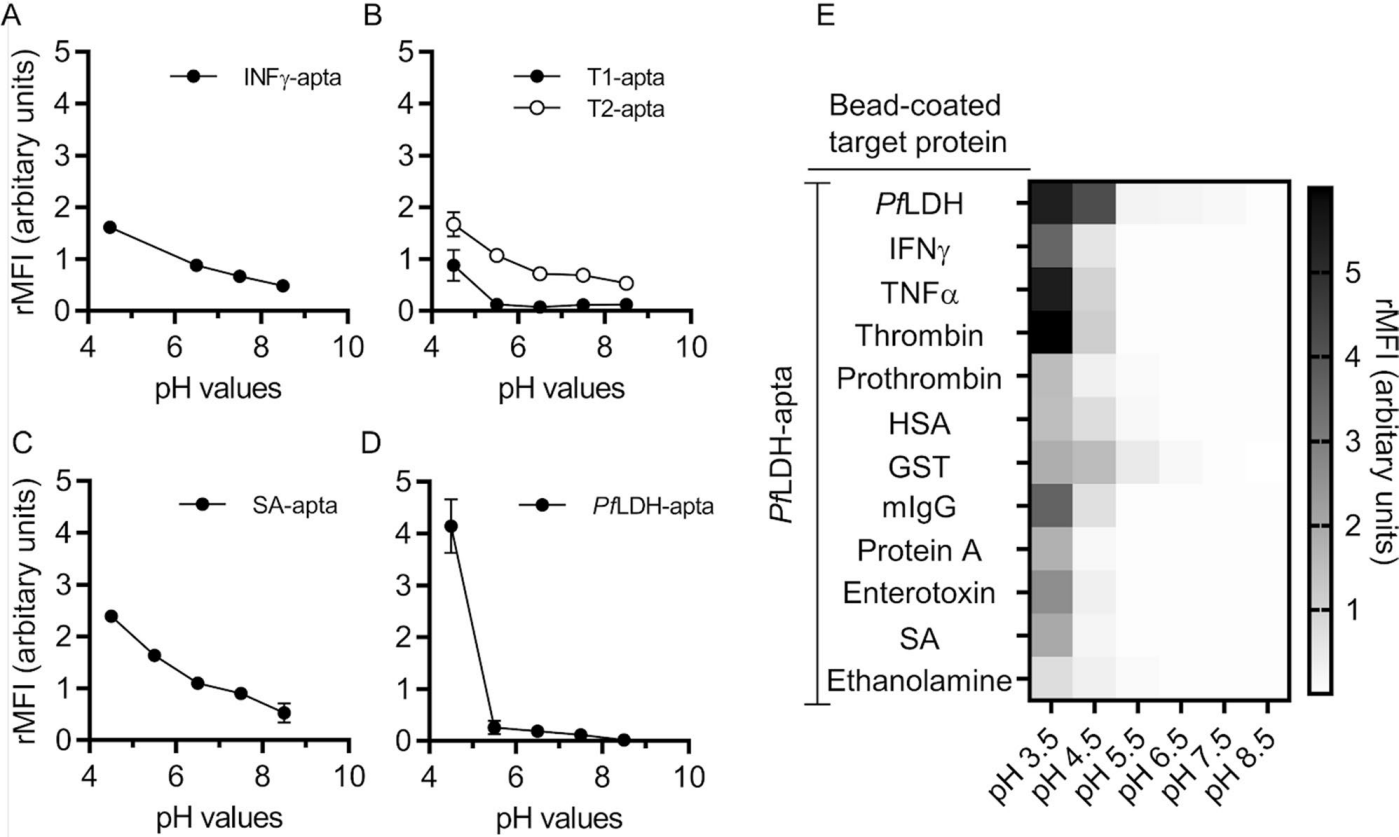
Influence of pH value on aptamer binding. Microbeads coupled with (**A**) IFNγ, (**B**) thrombin, (**C**) streptavidin and (**D**) *Pf*LDH were incubated with their fluorescence-labeled aptamer in binding buffers (= corresponding selection buffer) of varying pH values. (**E**) Binding capacity of *Pf*LDH-apta to non-target proteins immobilised on fluorescence-labelled microbeads under varying pH values. The binding of all aptamers to their targets were measured by quantifying the surface fluorescence of the microbeads using VideoScan technology. All data are represented as mean ± SD (n = 4).

At lower pH values (< pH 5) we observed higher aptamer binding, whilst at elevated pH values (> pH?) binding was suppressed (Fig. 4). However, at low pH all aptamers lost their specificity (PfLDH-apta Fig. 4E, remaining aptamers (Supplementary Fig. S4). In the range of the analysed pH values (pH 3.5–8.5) the aptamers are strongly negatively charged. At low pH the target proteins are considered to be strongly positively charged as indicated by their isoelectric point (Supplementary Table S3). This increase in the electrostatic binding affinity results in non-specific target binding.

Our data shows that aptamer binding does not strongly depend on the exact binding conditions as defined by the selection buffer during the SELEX procedure. We were able to alter the binding buffer composition considerably before we observed complete repression or non-specific binding.

### Divalent ions give rise to non-specific binding of aptamers

**Figure 5:**
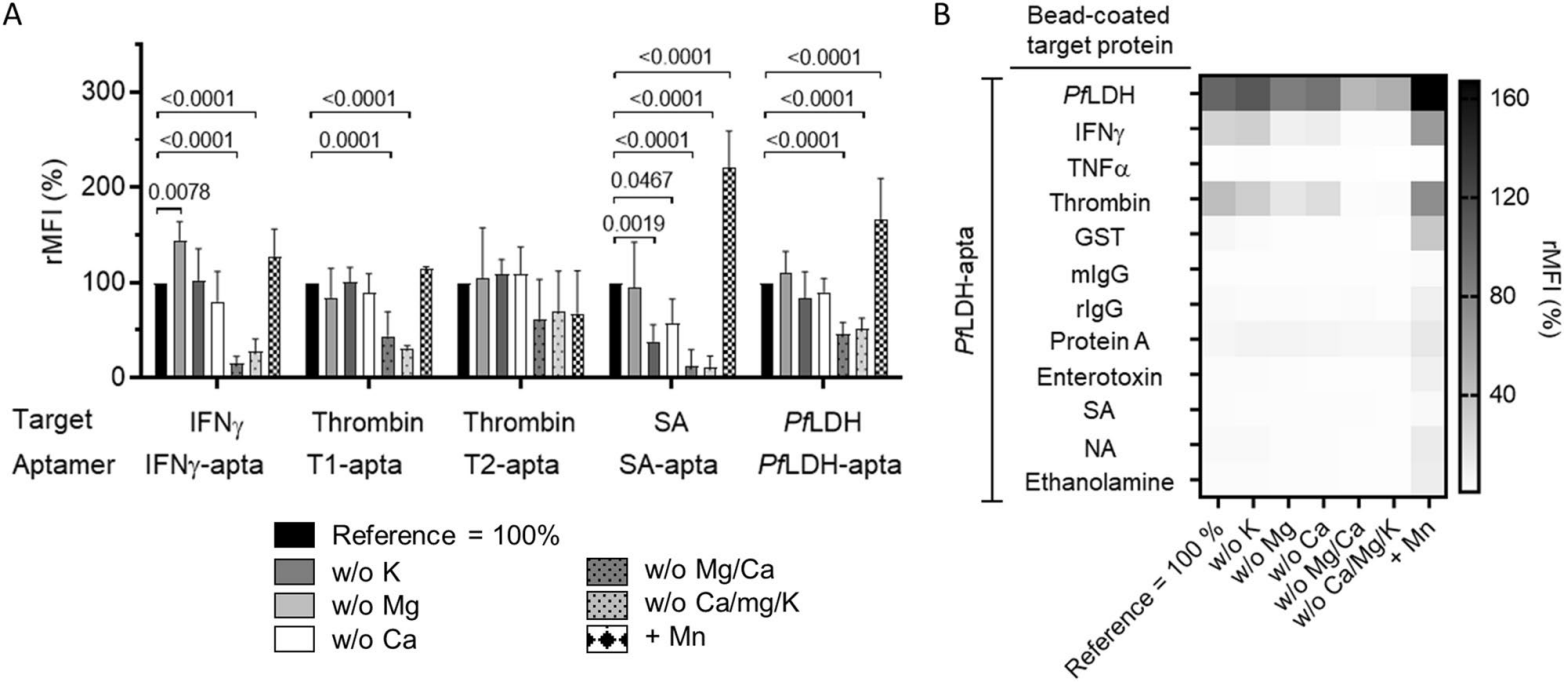
Influence of K^+^, Ca^2+^, Mg^2+^ and Mn^2+^ on aptamer binding. Fluorescence-labelled microbeads coupled with either IFNγ, streptavidin, thrombin or *Pf*LDH were incubated with indicated aptamers. (**A**) Streptavidin specific selection buffer functioned as a universal binding buffer with (+) or without (w/o) indicated components. (**B**) The Corresponding data of *Pf*LDH-apta to non-target proteins are shown in a heat map. For the other aptamers see supplementary information (Supplementary Fig. S5). All data represented as mean ± SD (n = 6).

The five investigated aptamers were mainly G-rich aptamer sequences that often form strong structures, like G-quadruplexes, in the presence of monovalent Na^+^ and K^+^ cations^27^. The removal of both Ca^2+^ and Mg^2+^ did lower the binding signal considerably (Fig. 5). This was expected for the SA-, T1- and T2-apta, as they were selected in the presence of these ions during the SELEX procedure. Independently we were not able to observe a significant K^+^-influence (Fig. 5A). In the case of *Pf*LDH-apta, selection was performed in a PBS buffer without Ca^2+^ and Mg^2+^. Unexpectedly, we observed a twofold increase with the addition of Ca^2+^/Mg^2+^ (Fig. 5A). With the exception of thrombin (eightfold) and IFNγ (sixfold), there was no observed increase of signal specificity control (Fig. 5B). We identified thrombin, IFNγ and also *Pf*LDH to be susceptible to non-specific binding in the presence of divalent cations (Fig. 5B and Supplementary Fig. S5A–D). We also observed that Mn^2+^ addition led to increased SA-apta binding (+ 100%, P < 0.001) and increased *Pf*LDH-apta binding (+ 50%, P < 0.001) (Fig. 5A). When the increasing signal of the specificity controls were also considered, signal amplification could only be achieved in the case of SA-apta, but not in case of P*f*LDH-apta.

### Modulation of aptamer binding by organic compounds

The organic compounds dimethylsulfoxide (DMSO) and tetramethylammonium chloride (TMAC) were added to further understand and explore aptamer/target binding stability. DMSO and TMAC are used in biochemistry to alter DNA melting points and hybridization kinetics as they impact A-T and G-C hybridization stabilities^28,29^. In order to amplify molecular interactions, PEG 8000 is added. To reduce non-specific interactions in assays we utilised Tween 20.

**Figure 6:**
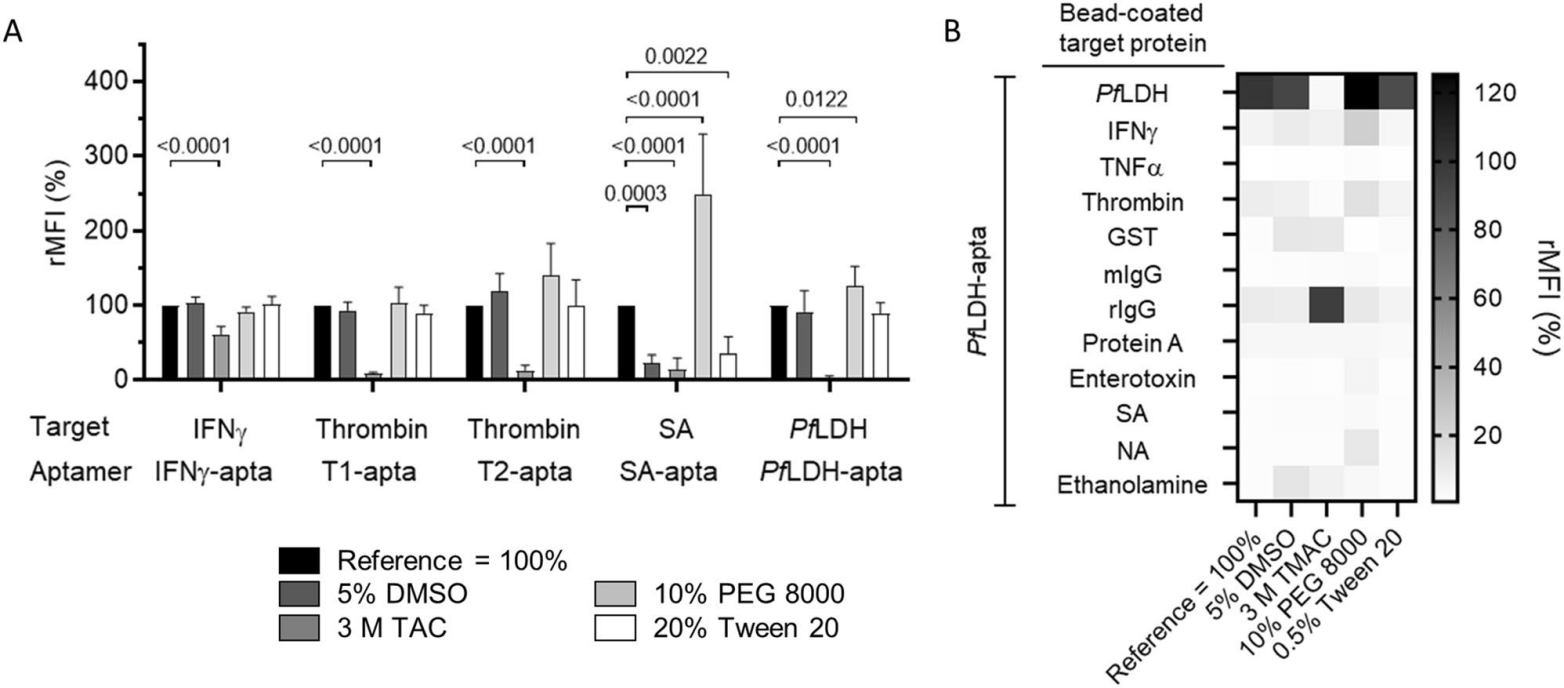
Influence of DMSO, PEG 800, TMAC and Tween 20 on aptamer binding. (**A**) Fluorescence-labelled microbeads coupled with either IFNγ, streptavidin, thrombin or *Pf*LDH were incubated with indicated aptamers. The streptavidin specific selection buffer functioned as a universal binding buffer supplemented with indicated components. (**B**) Heat map showing data of *Pf*LDH-apta to non-target proteins. The data of the other aptamers are shown in supplementary information (Supplementary Fig. S6). All data represented as mean ± SD (n = 6).

Addition of TMAC resulted in a strong decrease of binding of all aptamers to their target proteins (− 80 to − 100% in comparison to the reference) except for the binding of IFNγ-apta to its target protein IFNγ (− 40% in comparison to the reference) (Fig. 6A).

A relatively robust target/aptamer interaction was observed (excluding TMAC) (Fig. 6A), with the exception of SA-apta, where binding to streptavidin was reduced to ~ 20% in the presence of DMSO or Tween 20 (P < 0.001). PEG 8000 led to a doubling of the binding signal (P < 0.001), which was a real signal amplification since 12 out of 13 specificity controls were unaffected (Supplementary Fig S6A). The slight signal increase of *Pf*LDH-apta (+ 25%, P < 0.05) was accompanied by a corresponding increase in signal of the specificity controls, thus PEG 8000 did not improve the binding (Fig. 6B).

## Conclusion

Using aptamers originating from different SELEX screenings in one buffer system is possible after a simple screening process. Our data suggest that the aptamer binding is tolerant towards variations of ionic strength and pH value. Interestingly, an aptamer selection buffer is not necessarily the optimal buffer and could be easily improved, for example, simply by reducing the ionic strength. In contrast to the established detection methods, such as Surface plasmon resonance (SPR), flow cytometry and ELISA during SELEX^30^, we measured the aptamer-target binding with the fluorescence-based VideoScan technology. One advantage of our screening format wasthe simultaneous recording of several parameters (the simultaneous recording of several parameters (e.g. pH as well as ion gradients, chemical additives) under the same conditions in one functional test. Moreover, we covered a wide range of simultaneously investigated specificity controls. Multiplex screening technologies are of great interest when analysing biological samples with the use of aptamers, such as whole blood samples. We plan to address this challenge in future work.

A major advantage of this approach is the repeated measurement of samples and detection of artifacts, which enables the detection of aptamer-target binding in multiplex format in real time (Supplementary Fig. S5B). We believe that our developed microbead-based multiplex assay is suitable for systems with similar technical requirements. Especially in digital image analysis there are open source alternatives^31^ that can be adapted to analyze images taken with different fluorescence-based imaging platforms, making our approach applicable for any interested user.

## Methods

### Assay principle (VideoScan analysis)

All measurements of aptamer-target interactions were done with our in-house developed fully automatised multispectral inverse fluorescence microscopy platform, called VideoScan^9^, commercialised as Calaidoscan 100 (CS100) at Attomol GmbH (Germany) (Fig. 1). The VideoScan technology can be used to analyse cell assays, microbead assays, assays in solution and various other combinations. Applications include approaches for human diagnostics^32^, point of care testing^23^ and medical microbiology^33^. In our study, we covalently coupled a set of up to 17 different proteins by random amino-coupling individually to dye/size-encoded microbead populations and pooled them. Each microbead can be unambiguously assigned to a microbead population by its size and two fluorescent colours (different blue and green ratios), so that multiplexing is possible. As the dyes cannot leak from the microbeads, they form a stable reference system that allows the comparison of measured values between different studies. Aptamers were fluorescently labelled with Cy5 at the 5′-end of used aptamer sequences, the binding of aptamers causes fluorescence halo formation around the microbeads. This fluorescence halo was quantified by the VideoScan system and resulted in the parameter “referenced mean fluorescence intensity” (rMFI). We used 3D microbeads over a planar microarray, as microbeads have a larger analytical surface and allow rapid development of novel assays^34^. Our technology is not based on flow cytometry but used microbeads immobilised on the surface of a planar 96 well plate. Therefore, we were able to track signal changes in a time-dependent manner, which enabled us to record binding kinetics in a real-time format (Supplementary Fig. S5A). A microbead population with a higher rMFI had more bound fluorescence-labelled aptamers on the microbead surface.

IFNγ (#130-096-873) and TNFα (#130-096-017) were sourced from Miltenyi; rabbit anti-lacZ IgG (#A-11132) from MobiTEC; mouse IgG (#026502), protein A (#21181), anti-IFNγ IgG (P700), anti-TNFα IgG (P3001), biotinylated anti-HSA antibody (PA1-72057), APC-labeled streptavidin (SA 1005) and neutravidin (#31000) from ThermoFisher Scientific; thrombin (#ab62452) and anti-staphylococcal enterotoxin B IgG (#ab15898) from abcam; prothrombin (#BB004) from Binding Site; streptavidin from IBA (2-0203-010); protein G (P4689), staphylococcal enterotoxin B (S4881), human serum albumin (HSA) (SRP6182) and anti-Glutathione S-transferase IgG (anti-GST IgG) (G7781) from Sigma; anti-prothrombin IgG (11581-05011) from AssayPro; Fc-IgG1-fragment (011-00-008), Cy5-anti-rabbit IgG (111-175-144) and Cy5-anti-mouse IgG (515-175-003) from Dianova; glutathione S-transferase (GST) from Biotrend (00-001-200). All aptamer sequences were synthesised by biomers.net, microbead populations were from PolyAn.

### Bioinformatic analysis of the target proteins

For bioinformatic analyses, the *ProteinAnalysis* function, which is part of the Bio.SeqUtils package (https://biopython.org/docs/1.75/api/Bio.SeqUtils.html#) of Biopython (v. 1.75) under Python 3.7 was used to calculate characteristics of the protein based on a Python script^35^.

### Coupling of microbeads with proteins

Carboxylated PMMA microbeads (PolyAN GmbH, Berlin, Germany) were coated with proteins as described recently by Rödiger et al.^36^. Briefly, 300,000 microbeads were resuspended in 100 µL of 100 mM 2-(*N*-morpholino)ethanesulfonic acid (Mes, Sigma-Aldrich, Germany) buffer (pH 4.5) containing 25 mg mL^−1^ *N*-(3-dimethylaminopropyl)-*N*′-ethylcarbodiimide hydrochloride (EDC, Roth, Germany). The activated microbeads were incubated with a protein solution of 300 µg mL^−1^ in diluted PBS (2.5 mM Na-phosphate pH 7.4, 7.5 mM NaCl) for 3 h at 28 °C with continuous agitation to achieve covalent cross-linking via random amino-coupling. After washing three times with TBST (50 mM Tris/HCl, pH 7.4, 150 mM NaCl, 0.01% Tween 20), the protein-coated microbeads were ready for usage.

### Verification of successfully protein coupling onto the microbead surface

All binding data was measured by the use of proteins coupled to fluorescence-labelled microbeads. We verified that every protein was successfully coupled to microbeads by probing the microbeads with suitable detection probes, so that a signal could only be obtained when the expected protein was present on the microbead surface (Supplementary Fig. S1).

The following protocol was used::10 µL of microbead mixture was incubated at 25 °C for 1 h with 50 µL of TBST containing detection probes (For detailed information on which detection probe was used for each protein see Supplementary Table S2 in supplementrary information). For antibody incubation, a concentration of 1 µg mL^−1^ was used, for oligonucleotides we used 50 nM and for aptamers 500 nM. The microbeads were spun down by centrifugation and the supernatant was removed. The microbeads were washed three times with TBST and were either incubated with a secondary antibody (Supplementary Table S2) or directly subjected to VideoScan analysis.

### Generation of a multiplex microbead mixture

Microbead populations coupled to different proteins were chosen and mixed, so that a multiplex mixture was obtained that was composed of one microbead population presenting the aptamer target of interest. The remaining microbead populations represent the specificity controls. 10 µL of mixture contained approximately 500–1000 microbeads per population.

### Aptamer binding assay

A volume of 10 µL microbead suspension was mixed with 200 µL of binding buffer and the microbeads were spun down via centrifugation. Aptamer-specific selection buffers were either used directly or in a modified form according to the experimental requirements (Table 1). Every buffer was supplemented with at least 0.001% Tween 20 to prevent microbead attachment to the reaction tube walls and thereby prevent microbead loss during washing steps. After removal of the supernatant, 100 µL of 500 nM aptamer solution diluted in the respective binding buffer was added. The suspension was incubated at 25 °C with vigorous agitation for 1 h. Before VideoScan analysis unbound aptamers were removed by washing the microbeads three times with 200 µL of binding buffer.

### Fully automated image analysis (VideoScan analysis)

Microbeads were resuspended in 100 µL of binding buffer and transferred into cavities of a 96 well plate. The plate was placed onto the scanning stage of the VideoScan platform. After the microbeads had settled on the transparent bottom of the cavities, the measurement was started.

### Statistical analysis

All data were analysed in the *RKWard* integrated software environment (v. 0.7.1z + 0.7.2 + devel1^37^) and *Prism8* from Graph Pad Software (La Jolla, USA). *RKWard* and the *drc* package^38^ were used for curve fitting and calculation of the half-maximal values with a customised script. Out of eleven non-linear models the one with the lowest Akaike information criterion (AIC) was used. Statistical significance was analysed using one-way ANOVA by comparing the test group with the appropriate control groups.

## Supplementary Information

Supplementary Information.
