# Supplementary material for: A multiparametric fluorescence assay for screening aptamer–protein interactions based on microbeads
Source: Sci Rep. 2022 Feb 22;12:2961. doi: 10.1038/s41598-022-06817-0 (PMC8863788; doi:10.1038/s41598-022-06817-0)
Supplement: Supplementary file 1 — Supplementary Information. [file 41598_2022_6817_MOESM1_ESM.pdf]

Supplementary information

A Multiparametric Fluorescence Assay for Screening Aptamer-Protein Interactions Based on Microbeads

Carsten Schmidt[1], Anne Kammel[1,4], Julian A. Tanner[2], Andrew Kinghorn[2], Muhammad Moman Khan[1], Werner Lehmann[3], Marcus Menger[5], Uwe Schedler[6], Peter Schierack[1], Stefan Rödiger[1,4,*]

[1] Brandenburg Technical University Cottbus - Senftenberg, Faculty of Natural Sciences, Universitätsplatz 1, D-01968 Senftenberg, Germany

[2] The University of Hong Kong, 3/F., Laboratory Block, Faculty of Medicine, Department of Biochemistry, 21 Sassoon Road, Pokfulam, Hong Kong

[3] Attomol GmbH, Schulweg 6, 03205 Bronkow (Lipten), Germany

[4] Faculty of Health Brandenburg, Brandenburg University of Technology Cottbus - Senftenberg, Senftenberg, Germany

[5] Fraunhofer Institute for Cell Therapy and Immunology, Branch Bioanalytics and Bioprocesses (IZI-BB), Am Mühlenberg 13, D-14476 Potsdam, Germany

[6] PolyAn GmbH, Rudolf-Baschant-Straße 2, D-13086 Berlin, Germany

*corresponding author:

Email: stefan.roediger@b-tu.de

Phone: ++49 3573 85 93

This document contains supplementary information for the experimental section. We also provide additional aptamer binding data for the Result and Discussion part.

S-1: Table S1 with aptamer sequences and further characteristics

S-2: Binding data showing successful coupling (Figure S1)

S-3: Table S2 showing detection probes used for results in Figure S1

S-4: Table S3 with bioinformatic characteristics of proteins in Figure S1

S-5: Aptamer binding in dependence on aptamer concentration

S-6; Influence of heat on aptamer binding and binding kinetics

S-7: Supplementary results for Figure 4 in the manuscript (pH values)

S-8: Supplementary results for Figure 5 in the manuscript (K^+, Ca^{2+},Mg^{2+} and Mn^{2+})

S-9: Supplementary results for Figure 6 in the manuscript (DMSO, PEG 8000, TMAC,Tween 20)

S-10: References

Aptamer sequences and further characteristics

Table S1. Further characteristics of the used aptamers. Tm, melting temperature, as calculated using the nearest neighbor thermodynamics.

Aptamer	Sequence	mer	GC [%]	T_m [°C]
T1-apta	GGTTGGTGTGGTTGG	15	60.0	48.5
T2-apta	AGTCCGTGGTAGGGCAGGTTGGGGTGACT	29	62.1	71.3
IFNγ-apta	TGGGGTTGGTTGTGTTGGGTGTTGTGT	27	51.9	65.7
TNFα-apta	TGGTGGATGGCGCAGTCGGCGACAA	25	64.0	68.7
SA-apta	GGGAACGCACCGATCGCAGGTTTCCC	26	65.4	68.5
*PfL*DH-apta	CTGGGCGGTAGAACCATAGTGACCCAGCCGTCTAC	35	60.0	71.5
PA-apta	ATACCAGCTTATTCAATTAGCAACATGAGGGGGATAGAGGGGGTGGGTTCTCTCGGCTACAATCGTAATCA GTTAG	76	46.1	75.6
Entero-apta	GGTATTGAGGGTCGCATCCACTGGTCGTTGTTGTCTGTTGTCTGTTATGTTGTTTCGTGATGGCTCTAACTCTCCTCT	78	47.4	75.4
mIgG-apta	TAATACGACTCACTATAGCAATGGTACGGTACTTCCCCTCACCGGGTACCTGCCGCTCCCAAAAGTGCACGCTACT TTGCTAA	83	50.6	79.2

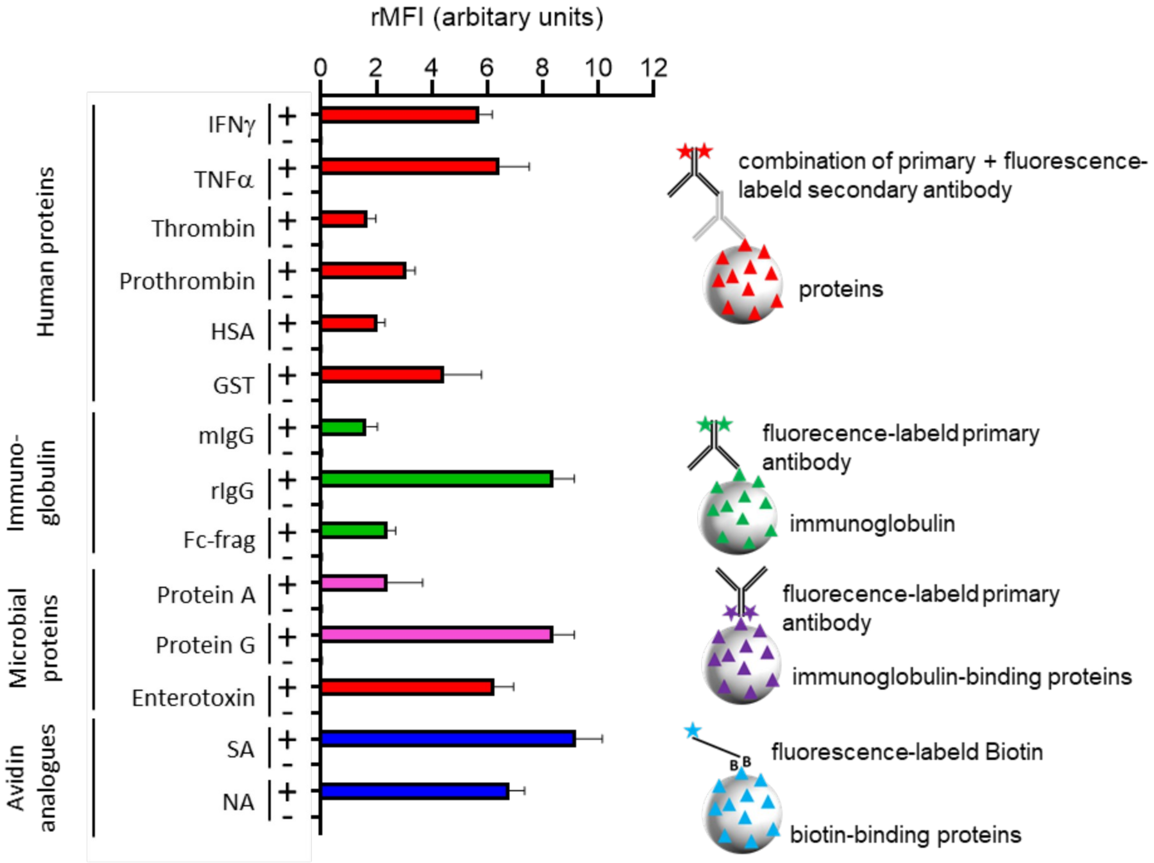

Figure S1. Verification of successfully coupled microbeads. Microbeads coupled with the indicated proteins (+) were incubated with appropriate fluorescence-labeled detection probes. As negative controls ethanolamine coupled microbeads were used (-). The presence of most proteins were verified via a primary antibody followed by a fluorescence-labeled secondary one (red), IgGs, protein A and protein G were detected directly via a fluorescence-labeled antibody (green, purple) and streptavidin (SA) as well as neutravidin (NA) were verified via a fluorescence-labeled biotinylated oligonucleotide (blue).

Detection probes used for results in Figure S1

Various detection probes had to be used. Although these detection probes are included in the experimental section, we decided to present additional tables with precise

information of each detection probes (Table S2) as well as target protein characteristics (Table S3).

Table S2. Detection probes used for verifying the presence of proteins on the microbead surface.

Protein	Detection via
IFNγ	rabbit anti IFNγ IgG, Cy5-labeled goat anti rabbit IgG
TNFα	rabbit anti TNFα IgG, Cy5-labeled goat anti rabbit IgG
prothrombin, thrombin	rabbit anti-prothrombin, Cy5-labeled anti rabbit IgG
GST	rabbit anti-GST IgG, Cy5-labeled anti rabbit IgG
GST	rabbit anti-GST IgG, Cy5-labeled anti rabbit IgG
mouse-IgG	Cy5-labeled sheep anti mouse IgG
rabbit Fc-IgG-fragment	Cy5-labeled Goat anti rabbit IgG
rabbit Fc-IgG-fragment	Cy5-labeled Goat anti rabbit IgG
protein A	Cy5-labeled Goat anti rabbit IgG
protein G	Cy5-labeled Goat anti rabbit IgG
Enterotoxin	rabbit anti-enterotoxin IgG, Cy5-labeled anti rabbit IgG
Neutravidin	Bis-biotinylated Atto647N-labeled oligo
Streptavidin	Bis-biotinylated Atto647N-labeled oligo

Bioinformatic characteristics of proteins in Figure S1

For bioinformatic analyses, the *ProteinAnalysis()* function of Biopython (v. 1.75) under Python 3.7 was used to calculate characteristics of the protein based on a Python script[1].

Table S3. Characteristics of target proteins. MW: Molecular weight of the protein. LA: length of amino acid sequence. AM: aromaticity value of a protein according to Lobry & Gautier 1994 (Lobry and Gautier, 1994). pI: Isoelectric point of the protein. *q* 5: charge of a protein at pH 5. *q* 10: charge of a protein at pH 10. ε_r: molar extinction coefficient assuming cysteines (reduced). ε_{cys}: molar extinction coefficient with cysteine residues (Cys-Cys-bond). II: instability index according to Guruprasad *et al.* 1990 (Guruprasad et al., 1990). An instability index larger than 40 indicates unstable proteins.

Protein	MW [kDa]	LA	AM	pI	q 5	q 10	ε_r	ε_{cys}	II
IFNγ	16.2	138	0.109	9.52	11.8	-6.06	11460	11460	29.2
TNFα	17.4	157	0.0828	7	5.45	-9.4	21430	21555	33.2
Ent B	28.4	239	0.142	8.25	11.6	-27.9	36790	36915	32.7
Prothrombin	65.3	579	0.095	5.24	6.15	-66.2	108290	109790	38.4
Thrombin	33.8	295	0.105	8.32	14.4	-22	65890	66390	31.8
HSA	66.5	585	0.0855	5.67	16.8	-86.6	32320	34445	38.8
*Pf*LDH	34.1	316	0.0506	7.12	13.2	-21.7	17420	17670	29.7
Protein A	48.6	441	0.0431	5.18	3.75	-45.5	7450	7450	54
Protein G	20.1	185	0.0919	4.37	-9.98	-30.5	28420	28420	7.85
Streptavidin	16.2	155	0.0903	6.72	3.42	-7.05	41940	41940	7.34
GST	25.6	219	0.123	5.9	7.01	-26.2	42860	43110	34.7
Fc-fragment	no sequence information available								
mIgG	no sequence information available								
rIgG	no sequence information available								

IFNγ, interferon γ (*human*, sp|P01579|24-161); TNFα, tumor necrosis factor α (*human*, sp|P01375|77-233); Ent B, enterotoxin B (*Staphylococcus aureus,* sp|P01552|28-266); prothrombin (*human*, sp|P00734|44-622), thrombin (*human*, sp|P00734|328-363 + sp|P00734|364-622); HSA, human serum albumin (sp|P02768|25-609); *Pf*LDH, lactate dehydrogenase, (*Plasmodium falciparum*, sp|Q27743|1-316); protein A (Staphylococcus aureus, sp|P38507|37-477); protein G (*streptococcus sp*, tr|Q54181|1-185); streptavidin (*streptomyces avidinii*, sp|P22629|25-183); GST, glutathione S-transferase (*Schistosoma japonicum*, sp|P08515|2-218).

Aptamer binding in dependence on aptamer concentration

Figure S5 shows that for all five aptamers that the binding to their immobilized targets has definitely plateaued at an aptamer concentration of 500 nM.

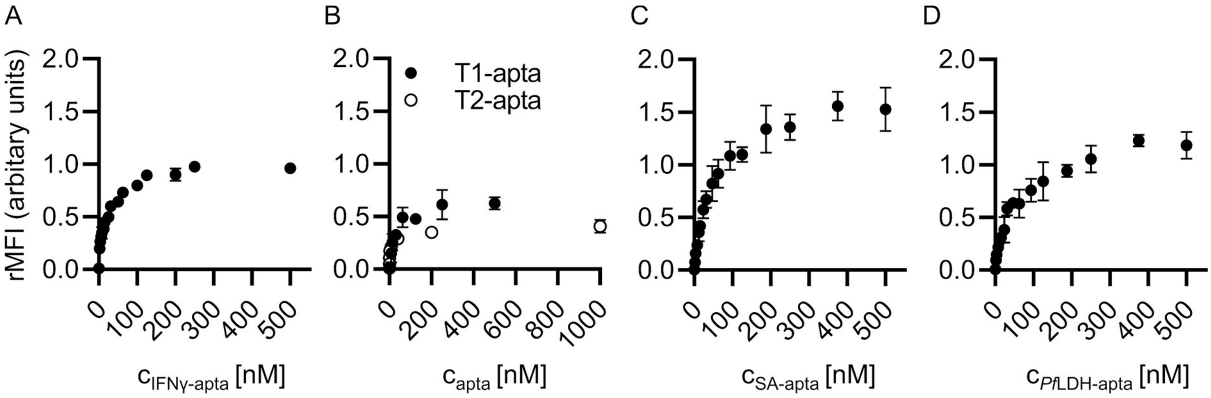

Figure S2. Aptamer binding curves. Microbeads coupled with interferon γ (A), thrombin (B), streptavidin (C), or PfLDH (D) were incubated with fluorescence labeled aptamers with the indicated concentrations. The aptamers were dissolved in their specific selection buffer. After 1 h at 25°C unbound aptamers were removed by washing and the aptamers that bound to the microbead surface were quantified with VideoScan technology as rMFI values. All data are represented as mean ± SD (n = 2).

Influence of heat on aptamer binding and binding kinetics

In addition, we also investigated the influence of heat to the aptamer-target interaction (Figure S3A) as well as the binding kinetics for two aptamers (Figure S3B).

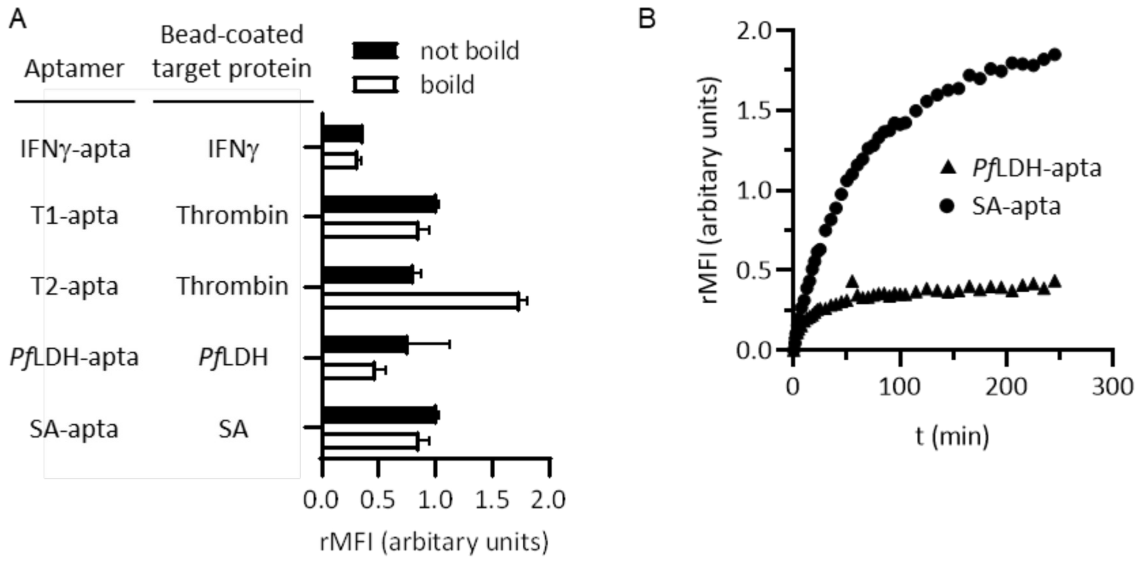

Figure S3. Influence of heat on aptamer binding and binding kinetics.. Microbeads coupled with the indicated proteins were incubated with fluorescence labeled aptamers. (A) Comparison of aptamer binding capacity with or without pretreatment of heating (95°C for 5 min) of the aptamer.(B) Depicted are the real-time kinetics of two aptamers (*Pf*LDH-apta towards *Pf*LDH and SA-apta towards SA) over a period of 240 min. The aptamers are dissolved in their specific selection buffer. All data are represented as mean ± SD (n = 2-4).

Supplementary results for Figure 4 in the manuscript (pH values)

In the manuscript, controls representing the aptamer specificity obtained for different pH values (Figure 4) are only shown for the *Pf*LDH-aptamer. Here, we show the corresponding aptamer-target binding data for the remaining four aptamers.

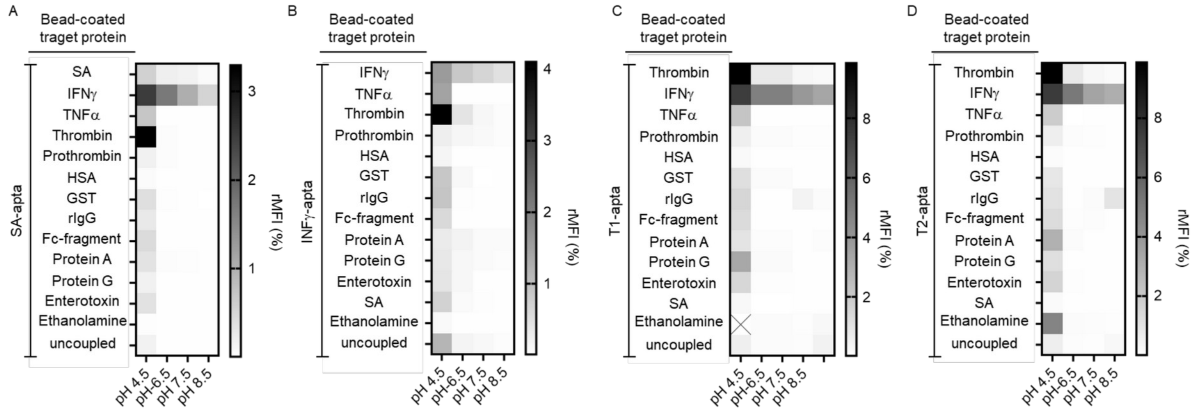

Figure S4. Influence of pH value on aptamer binding. Fluorescence-labeled microbeads coupled with indicated proteins were incubated with the appropriate fluorescence labeled aptamer (A-D) dissolved in its corresponding binding buffer, whose pH value was varied. After removal of unbound aptamer, the bound aptamer was measured by quantifying the surface fluorescence of the microbeads using VideoSan technology. Shown are mean values of four independent measurements.

S-8

Supplementary results for Figure 5 in the manuscript (K^+, Ca^{2+},Mg^{2+} and Mn^{2+})

In the manuscript, controls representing the aptamer specificity obtained for different ions and (Figure 5) are only shown for the *Pf*LDH-aptamer. Here, we show the corresponding aptamer-target binding data for the remaining four aptamers.

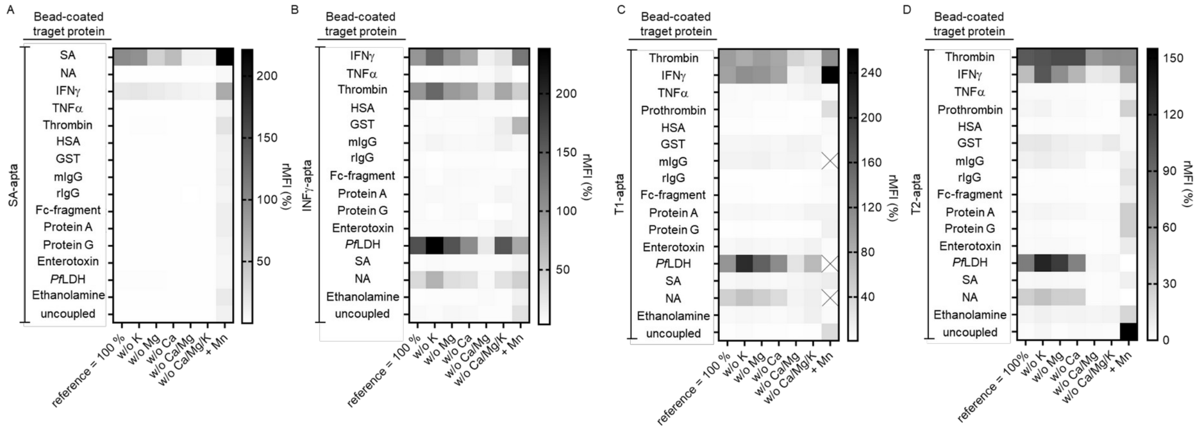

Figure S5. Microbeads coupled with indicated proteins were incubated with appropriate fluorescence labeled aptamers dissolved in a reference buffer (20 mM Tris/HCl pH 7.4, 100 mM NaCl, 5 mM KCl, 2 mM $MgCl_2$, 1 mM $CaCl_2$). (A-D) The relevance of K^+, Mg^{2+} and Ca^{2+} ions were checked by omitting these ions systematically. Shown are mean values of six independent measurements.

Supplementary results for Figure 6 in the manuscript (DMSO, PEG 8000, TMAC,Tween 20)

In the manuscript, controls representing the aptamer specificity obtained for organic compounds (Figure 6) are only shown for the *Pf*LDH-aptamer. Here, we show the corresponding aptamer-target binding data for the remaining four aptamers.

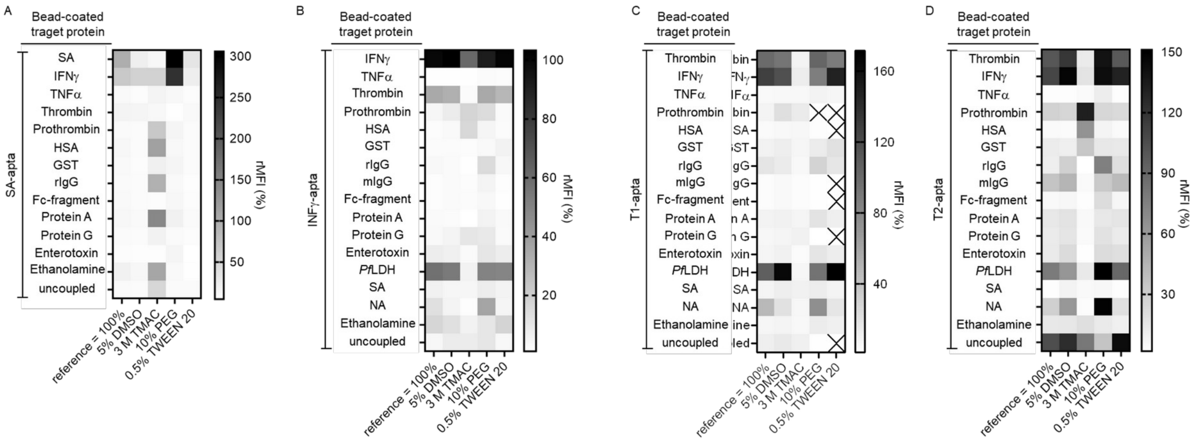

Figure S6. Microbeads coupled with indicated proteins were incubated with appropriate fluorescence labeled aptamers dissolved in a reference buffer (20 mM Tris/HCl pH 7.4, 100 mM NaCl, 5 mM KCl, 2 mM $MgCl_2$, 1 mM $CaCl_2$). (A-D) Represented is the influence of supplementation of chemical compounds to the reference buffer, such as DMSO, TMAC, PEG 8000 or Tween 20, on the aptamer binding. Shown are mean values of six independent measurements.

References

(1) Deutschmann, C.; Roggenbuck, D.; Schierack, P.; Rödiger, S. Autoantibody Testing by Enzyme-Linked Immunosorbent Assay-a Case in Which the Solid Phase Decides on Success and Failure. *Heliyon* **2020**, *6* (1), e03270. https://doi.org/10.1016/j.heliyon.2020.e03270.
